# Production vs. consumption drivers: Diagnosing urban and rural food N-footprint change in developed regions of eastern China

**DOI:** 10.1016/j.isci.2026.115698

**Published:** 2026-04-09

**Authors:** Chuanhe Xiong, Hengpeng Li, Askar Akida

**Affiliations:** 1 State Key Laboratory of Lake and Watershed Science for Water Security, Nanjing Institute of Geography and Limnology, Chinese Academy of Sciences, Nanjing 211135, China; 2 College of Resources and Environment, University of Chinese Academy of Sciences, Beijing 100049, China

**Keywords:** Environmental science, Aquatic science, Hydrology, Urban planning

## Abstract

Food nitrogen (N) footprint reflects the reactive N discharge resulting from meeting the basic food needs of the population, along with their environmental impacts. However, it remains unclear how the development of the food N footprint is reasonable. This paper refines the N-Calculator model and applies it to analyze food N footprints in developed regions of eastern China during 1990–2022. Our model simulations reveal two key findings: (1) The food consumption structure is in an imbalanced state and (2) reducing urban food N footprint—particularly its production-related footprints—is crucial for mitigating overall food N footprint growth in Jiangsu province, China. Our results highlight that optimizing residents’ food consumption structures and maintaining the minimum nutritional balance food demand, while meeting urban economic development needs through technological innovation and modern agriculture, and ensuring sufficient resources for non-point source pollution prevention, are essential to achieve a reasonable development of food N footprints.

## Highlights

• We enhanced the N-calculator model by incorporating food waste
• Food consumption structure is in an imbalanced state in Jiangsu province, China
• Reducing urban food nitrogen footprints is key to curbing food nitrogen footprint
• Curbing food nitrogen footprint relies on reducing its production-phase component

## Graphical abstract

**Figure:**
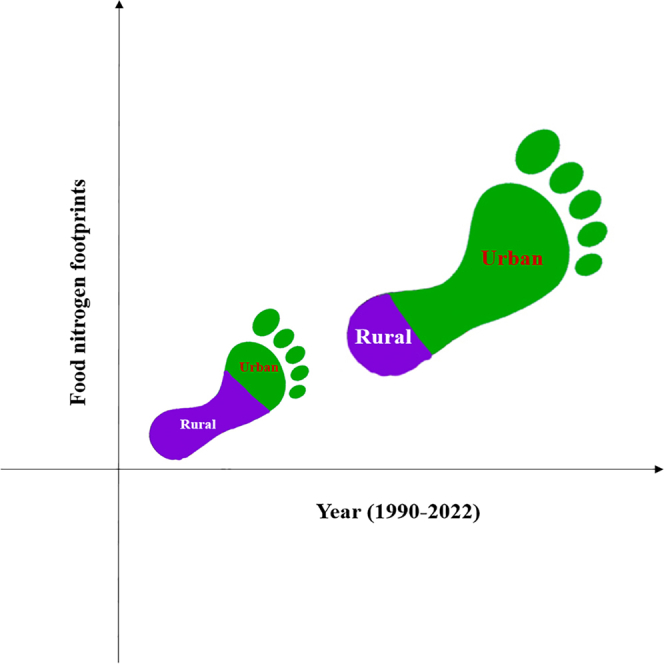

## Introduction

Nitrogen (N) in the form of reactive N (Nr; i.e., N in any form except N_2_) is not only a fundamental element and a key driver of terrestrial ecosystem functions but also a challenging pollutant to control.^1^ Excessive Nr discharge has disrupted the natural balance of the N biogeochemical cycle, leading to severe ecological and environmental issues such as surface water eutrophication, groundwater nitrate contamination, acid rain, soil acidification, greenhouse gas emissions, air pollution, and biodiversity loss.^2^ Thus, further research on Nr is imperative.

Due to disparities in non-economic factors such as natural conditions, geography, history, and culture, a persistent rural-urban divide exists. This gap is reflected not only in significant income inequality but also in distinct consumption structures and behaviors. Food consumption, a critical component of household expenditure,^3^^,^^4^ undergoes shifts in patterns and nutritional outcomes as societal development and economic growth drive rising demand. Notably, food systems contribute over 70% of total Nr discharge,^5^ constituting the largest anthropogenic Nr source and a key driver of global N pollution.^6^^,^^7^ Our prior research also indicated that dietary choices are the main contributing factor to Nr discharge.^8^ Thus, studying Nr dynamics in rural-urban food systems is essential for effective N pollution mitigation.

The food N footprint represents the amount of Nr released due to meeting a population’s basic food demand and its associated environmental impact.^5^^,^^8^^,^^9^ This topic remains a key focus in N footprint research.^10^ Current research has extensively investigated the magnitude and dynamics of food N footprints across multiple scales, with national-scale assessments being the most prevalent. Studies have quantified N footprints for various countries, including the United States,^5^ China,^11^ the Netherlands,^5^ Japan,^12^ the United Kingdom,^13^ Austria,^14^ and African nations.^15^^,^^16^ Research on urban food N footprints has quantified per capita values for multiple global cities, including Baltimore, USA,^17^ Toronto, Canada,^18^ and Shanghai, China.^19^ In existing studies, food N footprints are predominantly calculated using the N-Calculator model,^5^ integrated with regional population and per capita food consumption data to estimate regional N footprints. A review of existing research reveals several limitations. In survey-based studies, food consumption measurements typically exclude food waste, while studies using statistical data often equate food supply (which includes waste) with consumption. This practice results in double-counting in N footprint calculations. Why is there double-counting? The food N footprint comprises two components: the food consumption N footprint and the virtual N footprint. Virtual N consists of four components: N release during food production (such as the use of fertilizers and pesticides, pollution from livestock excretion, and so forth), food processing waste, spoiled food, and kitchen waste.^5^ Originally, food waste should ultimately be classified as kitchen waste, but it is instead classified as part of food consumption, ultimately leading to double counting of both the N footprint of food consumption and the N footprint of food production. Furthermore, research on rural residents’ food N footprint remains insufficient, and provincial-scale studies are particularly scarce.

In response to the problems existing in the food N footprint, such as repetitive calculation and insufficient comprehensive research on urban and rural areas, this paper aims to enhance the N-Calculator model by incorporating food waste, thereby resolving double-counting issues in N footprint calculations; Using the refined model, we assess the food N footprint of urban and rural residents in Jiangsu Province—a developed region in eastern China—examining total amounts, structural composition, and per capita levels. The findings aim to propose actionable pathways for reducing residents’ food N footprints and improving ecological sustainability in both urban and rural areas.

## Results

### Changing trends in per capita food consumption and per capita food nitrogen footprint of urban and rural residents

**Figure 1:**
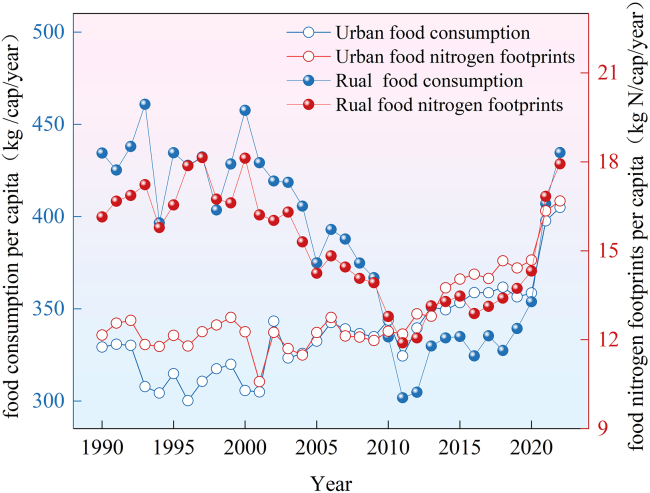
Variation of per capita food consumption and per capita food nitrogen footprints of Jiangsu residents during 1990–2022

Figure 1 illustrates trends in the food N footprint of urban and rural residents in Jiangsu Province (1990–2022). During the period from 1990 to 2010, the food N footprint of rural residents was higher than that of urban residents, with the gap gradually narrowing. From 2011 to 2020, the food N footprint of urban residents was higher than that of rural residents, with only a marginal difference. Since 2021, the food N footprint of rural residents has once again surpassed that of urban residents, with a widening disparity observed.

Meanwhile, variations in food consumption quantities correlate strongly with nearly identical trends in food N footprints (Figure 1). From 1990 to 2022, food consumption among urban residents showed a fluctuating upward trend—primarily driven by the increased consumption of vegetables, meat, and dairy products. Accordingly, the food N footprint rose steadily, peaking at 16.68 kg N/cap/yr in 2022. During this period, the decrease in the per capita grain consumption of urban residents led to a food N footprint of −0.33 kg N/cap/yr, accounting for −7.30% of the increase in the per capita food N footprint of urban residents; the increase in per capita meat consumption of urban residents resulted in an increase of 3.69 kg N/cap/yr in the food N footprint, accounting for 81.61% of the increase in the per capita N footprint of urban residents. The improvement in agricultural productivity (changes in virtual N factor (VNF)) reduced the urban residents’ food N footprint by approximately 1.18 kg N/cap/yr.

From 1990 to 2022, rural residents’ food consumption showed a U-shaped trend (Figure 1): initially declining (primarily due to reduced grain and vegetable consumption) before rebounding (driven by increased meat and dairy intake). Accordingly, the food N footprint first decreased then increased, peaking at 17.93 kg N/cap/yr in 2022. In 2011, the food N footprint of rural residents was the lowest, with a value of 11.90 kg N/cap/yr, marking a turning point. During 1990–2011, the decrease in the per capita grain consumption of rural residents led to a reduction of 5.70 kg N/cap/yr in the food N footprint, accounting for 134.13% of the reduction in the per capita food N footprint of rural residents; the increase in per capita meat consumption of rural residents led to an increase of 1.40 kg N/cap/yr in the food N footprint, accounting for −32.97% of the decrease in the per capita N footprint of rural residents. The improvement in agricultural productivity (the change in VNF) reduced the rural residents’ food N footprint by approximately 0.59 kg N/cap/yr. During 2011–2022, the decrease in per capita grain consumption of rural residents led to a food N footprint of −0.26 kg N/cap/yr, accounting for −4.31% of the increase in the per capita food N footprint of rural residents; the increase in per capita meat consumption of rural residents led to an increase of 4.00 kg N/cap/yr in the food N footprint, accounting for 66.24% of the increase in the per capita N footprint of rural residents. The improvement in agricultural productivity (the change in VNF) reduced the rural residents’ food N footprint by approximately 0.15 kg N/cap/yr.

**Figure 2:**
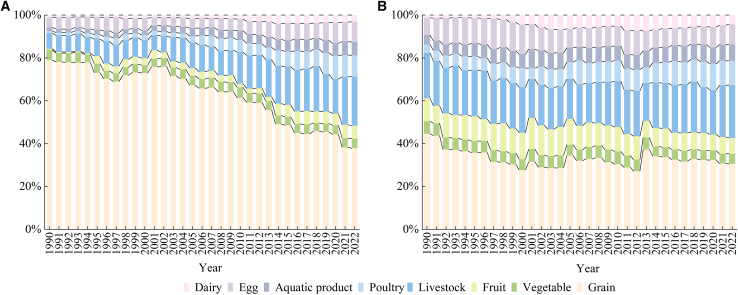
Structure of food nitrogen footprints in Jiangsu during 1990–2022 (A) Food nitrogen footprints per capita of rural residents. (B) Food nitrogen footprints per capita of urban residents.

Figure 2 illustrates structural changes in per capita N footprints across food categories from 1990 to 2022. From 1990 to 2022, rural residents’ diets remained grain- and vegetable-dominant, while urban residents maintained more balanced diets, with protein-rich foods (meat and dairy) accounting for 39–57% of their total food N footprint. During this period, protein-rich foods accounted for 16–52% of rural residents’ diets—showing growth but never surpassing 97% of urban levels. This disparity reflects varied dietary patterns and their impact on food N footprint dynamics. In terms of the overall food consumption structure of urban and rural residents in Jiangsu Province, the proportion of the N footprint of vegetarian foods represented by grains and vegetables has been continuously decreasing, while the proportion of the N footprint of fruit has relatively increased or fluctuated slightly. The proportion of the N footprint of meat products represented by livestock and poultry meat and aquatic products gradually increased and then gradually stabilized, rising from 30% (urban) and 10% (rural) to 44% (urban) and 39% (rural), respectively. The proportion of non-staple food consumption, such as eggs and dairy products, has also gradually increased, rising from 9% (urban) and 6% (rural) to 18% (urban) and 13% (rural), respectively. It can be seen that with the improvement of living standards, urban and rural residents in Jiangsu Province tend to consume high-N livestock and poultry meat and aquatic products. Their choices of vegetarian food are also more inclined toward nutrient-rich fruits and vegetables. At the same time, they have increased the consumption of high-protein side dishes. Daily food consumption is no longer focused on merely satisfying hunger but is progressively shifting toward seeking high-quality and refined food.

Analysis of N footprints in food production and consumption reveals distinct trends between urban and rural residents in Jiangsu Province. Among urban residents, the food N footprint has remained stable, with approximately 80% attributed to production and 20% to consumption (Figure S1). In contrast, rural residents food N footprint experienced significant shifts: the production share rose from 73.45% (1990) to 78.42% (2022), while the consumption share fell from 26.55% to 21.58% (Figure S1). Overall, whether in urban or rural areas, the proportion of food production N footprint to food N footprint is over 70%. Consequently, reducing the overall food N footprint hinges primarily on lowering the food production footprint.

### Changes in the total food nitrogen footprint of urban and rural residents

**Figure 3:**
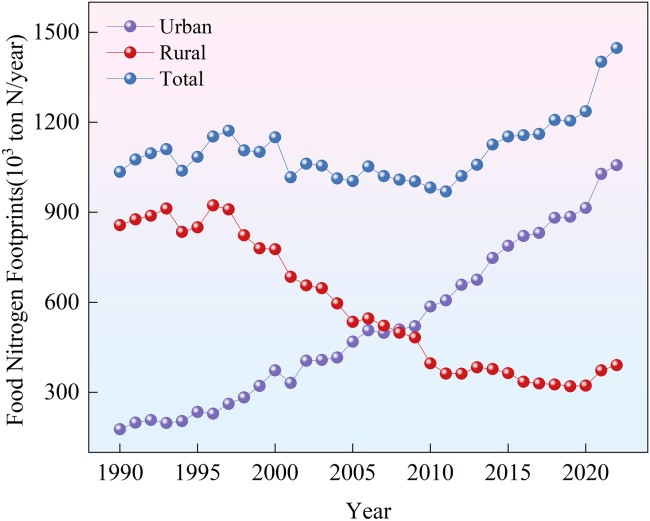
Variation of food nitrogen footprints of Jiangsu during 1990–2022

Changes in both per capita food N footprint and regional permanent population collectively drive the total food N footprint variation. In 1990, Jiangsu Province’s urban permanent population was 14.59 million. By 2022, it had risen to 63.37 million, marking a 434.34% increase compared to 1990. The total food N footprint of urban residents in Jiangsu Province increased by 879.7 × 10^3^ tons (N) in 2022 compared to 1990, reaching a peak of 1057.2 × 10^3^ tons N/yr in 2022, showing a rapid upward trend overall (Figure 3). The total food N footprint of rural residents ranged from 320.2 × 10^3^ tons N/yr (minimum in 2019) to 923.5 × 10^3^ tons N/yr (maximum in 1996), exhibiting an overall decreasing trend (Figure 3).

Overall, the food N footprint in Jiangsu Province increased from 1034.5 × 10^3^ tons N/yr in 1990 to 1447.8 × 10^3^ tons N/yr in 2022, showing an upward trend with an annual growth rate of 1.06% (Figure 3). Through decomposition analysis, it can be observed that with other factors remaining constant, the urban population growth between 1990 and 2022 contributed to an increase of 593.3 × 10^3^ tons N in the food N footprint, accounting for 143.55% of the total increase. The combination of urban population growth and an increase in per capita N footprint of urban residents led to an additional 879.74 × 10^3^ tons N of food N footprint, representing 212.85% of the total increase (This percentage contribution represents “relative contribution to net change”). Conversely, the decrease in rural population resulted in a reduction of 505.36 × 10^3^ tons N in the food N footprint, accounting for −122.27% of the total increase. The combination of rural population decrease and an increase in per capita N footprint of rural residents led to a decrease of 466.41 × 10^3^ tons N in food N footprint, representing −112.85% of the total increase. Over the past 32 years, while Jiangsu Province’s rural food N footprint has decreased, its urban food footprint (with a larger population) has grown consistently. This suggests that urban per capita food N footprints are the primary driver of Jiangsu’s overall food N footprint. Consequently, reducing urban per capita levels presents the key to curbing the province’s growing food N footprint.

## Discussion

### Differences in the food nitrogen footprint of urban and rural residents before and after model improvement

By improving the model to account for food waste rates, we resolved the issue of double-counting in food N footprint calculations. The per capita food N footprint for urban residents decreased by 0.73–0.92 kg N/cap/yr (among them, the meat and grains decreased by 0.29–0.39 kg N/cap/yr), representing a 4.55–7.12% reduction; for rural residents, the decrease was 0.62–0.93 kg N/cap/yr (among them, the meat and grains decreased by 0.41–0.57 kg N/cap/yr), a 3.67–6.16% reduction. The significant difference between pre- and post-improvement data highlights the flaws in the original algorithm and underscores the importance of incorporating food waste rates in model refinements.

### Comparison of the food nitrogen footprint of urban and rural residents in Jiangsu Province with that in other regions

In 2022, the per capita food N footprint of urban residents in Jiangsu Province was 16.68 kg N/cap/yr, compared to 17.93 kg N/cap/yr for rural residents. These values represented 74.60% (urban) and 80.19% (rural) of Jiangsu’s total per capita N footprint,^8^ and 51.44% (urban) and 55.00% (rural) of China’s per capita N footprint,^20^ confirming the food N footprint as the primary contributor to N footprints. A cross-regional comparison (Table 1) revealed that Jiangsu—a developed eastern Chinese region—exhibited lower urban and rural food N footprints than Western countries (e.g., Europe and North America) but higher levels than many developing nations. Jiangsu’s food N footprint growth follows the universal pattern of “economic development → dietary upgrading → N footprint increase.” It also demonstrates the effectiveness of environmental protection strategies in reducing the food N footprint.

**Table 1:** Comparison of the food nitrogen footprint of urban and rural residents in Jiangsu Province with that in other regions

Regions	Food nitrogen footprints	Source
Jiangsu-urban	16.68	This paper
Jiangsu-rural	17.93	This paper
China-urban	19.33	Lu et al.^21^
China-rural	19.69	Lu et al.^21^
USA	27.00	Leach et al.^5^
UK	18.00	Stevens et al.^13^
Japan	25.6	Shibata et al.^12^
Sweden	12.10	Einarsson et al.^22^
Thailand	11.08	Mungcharoen et al.^23^
India	11.40	Oita et al.^24^
Tanzania	8.80	Hutton et al.^15^

### The reasonable food nitrogen footprint range for rural and urban residents in Jiangsu Province

Through a comparison of the per capita food consumption of urban and rural residents in Jiangsu Province with the balanced per capita food consumption recommended by the Dietary Guidelines for Chinese Residents (the “nutritional balance” targets are for the average adult),^25^ it was found that urban residents consumed reasonable amounts of grains, vegetables, and eggs, but their meat consumption was higher than required for China’s urban and rural residents’ nutritional balance (Table 2). Consumption of fruits, aquatic products, and dairy products was lower than recommended (Table 2). For rural residents, vegetable and egg consumption was reasonable (Table 2); grain consumption remained high, but as the rural economy developed and the income gap between urban and rural residents narrowed, rural residents’ dietary habits increasingly aligned with those of urban residents, and grain consumption became more reasonable (Table 2). However, meat consumption experienced a shift from lower to reasonable and then to higher (Table 2). Consumption of fruits, aquatic products, and dairy products was also lower than required for China’s urban and rural residents’ nutritional balance (Table 2). According to the balanced per capita food consumption recommendations from the 2016 Chinese Dietary Guidelines, the calculated N footprint lies between 16.48 kg N/cap/yr and 27.19 kg N/cap/yr. In 2022, the food N footprint of urban residents was 16.68 kg N/cap/yr, and that of rural residents was 17.93 kg N/cap/yr, both falling within the balanced range for per capita food N footprints among Chinese residents. However, the consumption structure was not optimal. Therefore, optimizing residents’ food consumption structure and maintaining minimal nutritional balance food needs can, to a certain extent, inhibit the unlimited growth of personal food N footprints. This is of utmost importance for Jiangsu Province’s strategy of achieving low-N development.

**Table 2:** Annual mean food consumption and nutrient balance of Chinese residents and residents in Jiangsu Province

Food category	Annual mean food consumption for nutrient balance/kg	Food consumption of urban residents/kg	Food consumption of rural residents/kg
Grain	91.3–146.0	74.1–120.5	133.9–292.0
Vegetable	109.5–182.5	104.7–128.8	86.9–133.0
Fruit	73.0–146.0	36.1–59.2	3.2–46.8
Meat	18.3–27.3	24.5–50.0	11.0–49.0
Aquatic product	27.4–46.5	10.7–23.9	5.4–21.1
Egg	9.1–18.3	7.5–14.8	6.0–15.9
Dairy	109.5–182.5	3.7–23.7	3.4–12.3

### Policy recommendations for reducing the food nitrogen footprint of urban and rural residents in Jiangsu Province

1. To optimize residents’ food consumption structure while maintaining minimum nutritional requirements, the focus should be on: reducing the intake of high-N foods such as pork, beef, mutton, and poultry, and increasing the consumption of fruits, aquatic products, and dairy products. Tax exemption policies for plant-based food producers could stimulate bean production and consumption. This approach aligns with initiatives such as the EU’s strategy to reduce meat consumption by 15% within a decade.^26^
2. To reduce N discharge in food production, a “N compensation” mechanism could be implemented, or dedicated fiscal support could be provided for modern agricultural development. Such measures ensure that technological innovation and modern agriculture meet urban economic development needs while providing adequate resources for non-point source pollution prevention. Only then can there be a substantial reduction in urban residents’ food N footprint, ultimately achieving regional coordinated development and synergistic N discharge reduction outcomes.
3. To reduce food waste and enhance N utilization efficiency in food consumption, thereby decreasing environmental N discharge. Specific measures include formulating a “Household Cooking Guide Manual” to guide residents in reasonably calculating the amount of grain and oil used per meal based on different circumstances; reducing food waste in canteens and restaurants and encourage people to take leftovers home; promoting the extension of cold chain logistics to rural areas to slow down the rate of spoilage of fresh food; and increasing efforts to raise awareness about the importance of food conservation.

### Limitations of the study

This study calculated the food N footprint of urban and rural residents in Jiangsu Province, accounting for food waste in consumption. Although this study employs the VNF for different years in Jiangsu Province, variations in both natural factors (e.g., climate, soil, and precipitation) and socioeconomic factors (e.g., feeding practices and waste management) may significantly influence regional VNF values of food. To improve accuracy, future research should conduct localized VNF calculations and comparative analyses across representative regions.

## Resource availability

### Lead contact

Further information and requests for resources should be directed to and will be fulfilled by the lead contact, Chuanhe Xiong (chhxiong@niglas.ac.cn).

### Materials availability

No new materials were generated by this work.

### Data and code availability

• This study analyzes existing, publicly available data. These accession numbers for the datasets are listed in the key resources table.
• This study does not report original code.
• Any additional information required to reanalyze the data reported in this study is available from the lead contact upon request.

## Acknowledgments

This work was supported by the International Partnership Program of the 10.13039/501100002367Chinese Academy of Sciences (045GJHZ2024045FN), 10.13039/501100001809National Natural Science Foundation of China of China (42001133), the Basic Research Program of Jiangsu (BK20250110), and THE 10.13039/501100012166National Key R&D Program of China (2023YFD1702101).

## Author contributions

C.X.: data curation, methodology, writing – original draft, and validation. H.L.: writing- reviewing and editing. A.A.: visualization.

## Declaration of interests

The authors declare no competing interests.

## STAR★Methods

### Key resources table

**Table:** 

REAGENT or RESOURCE	SOURCE	IDENTIFIER
**Deposited data**		

Food waste information	China Health and Nutrition Survey	https://chns.cpc.unc.edu/data/datasets/.
Food supply information	Statistical Year books	http://www.jiangsu.gov.cn/col/col84736/index.html
Nitrogen content of different foods	Literature	https://doi.org/10.1016/j.envint.2016.08.012
Jiangsu’s virtual nitrogen factor	Literature	https://doi.org/10.1016/j.jclepro.2023.136011

**Software and algorithms**		

Origin2024	OriginLab	www.originlab.com

### Method details

#### Food nitrogen footprints calculation

This study examines the food nitrogen footprint among Jiangsu Province residents, encompassing discharge of Nr across the entire food life cycle (production → primary processing → transportation → secondary processing → consumption). We employ an improved N-Calculator model to quantify the food nitrogen footprint. The food nitrogen footprint (FP_T_) comprises two components: the food consumption nitrogen footprint (FP_C_) and the food production nitrogen footprint (FP_P_), that is:

(Equation 1) $$FP_{T}=FP_{C}+FP_{P}$$

The food consumption nitrogen footprint is calculated using two key parameters: per capita food consumption and nitrogen content in food.

It should be noted that official food consumption statistics include food waste, representing per capita food supply rather than actual consumption. To estimate true consumption, we adjusted the data using food waste rates, yielding the following food nitrogen footprint calculation formula:

(Equation 2) $${FP_{C}=\sum }_{i}^{n}{FPc}_{i}=\sum_{1}^{n}W_{i}\;\left(1-r_{i}\right)N_{i}$$

*W*_*i*_ represents the per capita food supply of the i-th type of food, *r*_*i*_ represents the waste rate of the i-th type of food, and *N*_*i*_ indicates the nitrogen content of the i-th type of food. Regarding the food nitrogen footprint calculation in this study, we assume all dietary nitrogen intake by adults is eventually excreted into the environment through feces and urine.^4^ Residents in Jiangsu Province primarily consume a diet consisting of grains, vegetables, fruits, and animal products including pork (dominant meat), poultry, aquatic products, eggs, and dairy products. The nitrogen contents and waste rates for different food categories are detailed in Tables S1 and S2.

The food production nitrogen footprint represents virtual nitrogen, which encompasses all nitrogen losses occurring during agricultural processes - including fertilizer application, livestock/poultry farming, and food processing - but not directly consumed by humans. The food production nitrogen footprint of the i-th type of food (FPp_i_) is calculated by multiplying the virtual nitrogen factor of the i-th type of food (EF_i_) (Table S3) with the food consumption nitrogen footprint of the i-th type of food, based on the relationship between food-specific virtual nitrogen and consumed nitrogen.

(Equation 3) $${FP_{P}=\sum_{i}^{n}FPp}_{i}=\sum_{i}^{n}{FPc}_{i}\times {EF}_{i}$$

To establish clear system boundaries and prevent double-counting, virtual nitrogen calculations generally exclude nitrogen losses resulting from energy consumption involved in food production processes. Because these are typically attributed to the energy sector. Accordingly, in the calculations of this paper, virtual nitrogen excludes discharge of nitrogen from energy consumption during food production, processing, and transportation.

#### Data sources

This study analyzes the food nitrogen footprint of urban and rural residents in Jiangsu Province (1990–2022). Per capita supply data for major food categories - including grains, vegetables, fruits, meat (mainly pork), poultry, aquatic products, eggs and dairy products - were obtained from the “Jiangsu Statistical Yearbook” (1991–2023) and “Jiangsu Statistical Bulletin” (1991–2023). Food nitrogen parameters were sourced from published studies on Jiangsu.^11^^,^^27^ The waste amounts of different types of food in urban and rural areas of Jiangsu Province provided by CHNS (China Health and Nutrition Survey) and some literature.^28^^,^^29^^,^^30^

### Quantification and statistical analysis

Statistical analyses were performed using Excel. Figures 1, 2, and 3 were produced using Origin 2024.
