# Supplementary material for: Production vs. consumption drivers: Diagnosing urban and rural food N-footprint change in developed regions of eastern China
Source: iScience. 2026 Apr 9;29(5):115698. doi: 10.1016/j.isci.2026.115698 (PMC13157049; doi:10.1016/j.isci.2026.115698)
Supplement: Document S1. Figure S1 and Tables S1–S3 [file mmc1.pdf]

iScience, Volume *29*

Supplemental information

Production vs. consumption drivers: Diagnosing urban and rural food N-footprint change in developed regions of eastern China

Chuanhe Xiong, Hengpeng Li, and Askar Akida

Table S1 the nitrogen content of different foods[1]

Type/year	Nitrogen content (g/kg)
Grain	13.60
Vegetable	2.10
Fruit	2.00
Livestock meat	17.70
Poultry	19.00
Aquatic product	14.10
Eggs	18.30
Dairy	5.50

Table S2 the food waste rate[2-5]

Table S2-1 the food waste rate of urban residents in Jiangsu

Food waste ratio	Grain	Vegetable	Fruit	Livestock meat	Poultry	Aquatic product	Eggs	Dairy
1990	3.62	6.95	1.95	9.27	8.05	9.82	7.18	7.16
1991	3.62	6.83	2.52	9.27	8.71	9.63	5.43	6.66
1992	4.38	7.59	2.05	9.48	8.29	9.14	4.88	6.91
1993	4.78	8.36	2.37	9.57	9.46	9.85	5.50	8.98
1994	4.76	8.27	2.12	10.16	7.74	9.04	5.10	8.47
1995	4.66	7.79	1.99	9.48	7.66	7.96	5.13	7.40
1996	4.60	7.58	2.11	9.00	7.72	7.26	4.98	6.64
1997	4.93	7.28	1.63	9.50	5.46	6.53	4.12	5.51
1998	5.06	7.76	1.64	9.35	6.24	6.41	4.54	4.68
1999	5.25	7.97	1.96	9.31	6.25	6.45	4.42	3.03
2000	5.28	8.51	1.44	11.34	5.46	9.52	5.41	3.46
2001	5.46	8.42	1.47	12.34	8.33	9.28	5.74	3.25
2002	5.29	8.23	1.43	10.87	9.04	14.56	5.60	2.24
2003	5.48	8.50	1.58	10.78	8.96	14.74	5.63	1.94
2004	6.04	8.09	2.57	11.00	9.88	13.36	4.40	1.18
2005	4.54	8.02	2.33	9.88	9.11	13.13	4.81	1.09
2006	4.29	7.14	2.20	8.80	7.07	11.01	4.58	0.94
2007	4.45	7.07	2.37	9.81	8.69	10.33	4.77	0.97
2008	4.46	7.10	2.69	9.68	8.65	10.88	4.69	1.10
2009	5.49	6.77	2.54	9.44	8.56	11.06	4.54	1.07
2010	5.54	6.56	2.50	8.89	8.36	11.13	4.51	1.04

2011	5.73	6.64	2.70	8.77	8.00	11.33	4.05	1.07
2012	5.85	6.59	2.45	8.27	7.62	10.65	3.80	1.01
2013	4.40	6.38	3.49	9.92	10.14	9.59	4.70	1.00
2014	4.44	7.16	2.90	7.81	7.73	9.66	4.84	0.99
2015	4.40	7.13	2.82	7.63	7.21	9.47	4.55	1.08
2016	4.42	7.03	2.69	7.64	6.45	9.19	4.43	1.12
2017	4.49	6.76	2.49	7.41	6.98	9.03	4.24	1.13
2018	4.13	7.06	2.41	6.81	6.59	9.02	4.25	1.16
2019	4.16	6.93	2.30	7.44	5.89	8.17	4.10	1.17
2020	4.05	6.73	2.35	7.39	5.65	8.23	3.55	1.10
2021	3.93	6.07	2.15	5.94	5.46	7.74	3.38	1.20
2022	3.83	5.68	2.20	5.63	5.40	7.45	3.18	1.27

Table S2-1 the food waste rate of rural residents in Jiangsu

Food waste ratio	Grain	Vegetable	Fruit	Livestock meat	Poultry	Aquatic product	Eggs	Dairy
1990	2.36	8.07	4.14	8.12	9.30	11.44	8.56	5.07
1991	2.50	9.70	4.72	7.93	10.03	11.80	7.59	5.07
1992	2.73	10.12	5.54	8.49	9.84	13.67	7.84	5.47
1993	2.99	10.25	6.55	8.53	10.69	14.39	8.92	6.65
1994	2.95	12.63	5.65	9.20	8.46	12.10	8.24	5.73
1995	3.18	11.67	3.32	8.92	9.96	11.18	7.60	5.54
1996	3.05	11.84	3.28	8.28	10.90	11.70	6.61	5.25
1997	3.08	11.65	3.32	8.48	8.28	12.36	5.90	4.51
1998	3.04	11.98	3.11	9.66	9.21	14.07	8.40	4.89
1999	3.11	12.05	3.53	9.96	12.73	14.25	9.38	4.95
2000	2.97	11.58	6.14	9.41	12.46	16.45	8.11	6.49
2001	3.03	11.41	5.12	9.70	12.44	15.67	10.18	6.11
2002	2.97	11.22	6.91	8.75	12.43	13.85	10.16	5.91
2003	3.04	10.90	6.45	9.79	11.18	12.76	9.31	5.81
2004	3.33	10.55	1.82	10.65	16.25	16.74	8.35	4.61
2005	3.51	10.56	1.62	10.70	12.63	13.86	8.37	3.72
2006	2.76	10.79	6.10	10.35	12.35	14.79	9.29	5.23
2007	2.76	10.37	6.16	11.53	11.01	13.40	10.02	5.09
2008	2.82	10.01	6.39	11.54	9.86	13.54	8.01	4.89
2009	2.86	9.59	5.64	12.71	11.17	15.55	8.40	4.12
2010	3.07	8.94	7.22	10.81	11.45	14.24	8.55	3.83
2011	3.23	9.47	8.18	10.34	11.99	14.40	8.73	3.55
2012	3.18	9.51	6.83	10.19	12.61	14.47	8.07	3.52
2013	3.18	9.44	7.98	8.65	10.95	12.14	6.85	3.08
2014	3.54	9.60	3.02	8.45	10.69	11.47	6.57	2.40
2015	3.50	9.57	2.94	8.17	10.71	11.13	5.92	2.50
2016	3.75	9.38	2.44	8.16	9.41	10.64	6.01	2.57
2017	3.70	8.73	2.20	7.83	10.04	10.42	5.73	2.48
2018	3.46	9.58	2.41	7.14	9.94	10.79	5.91	2.44
2019	3.42	8.95	2.28	8.31	7.83	9.59	4.99	2.54

2020	3.36	8.35	2.23	8.64	6.94	9.24	4.16	2.45
2021	3.47	7.44	1.82	5.64	6.52	7.69	3.87	2.39
2022	3.34	6.82	1.85	5.22	6.37	7.30	3.56	2.37

Note: The category-specific food waste rates were calculated by dividing the waste quantities for different food types (obtained from the China Health and Nutrition Survey [CHNS] and relevant literature) by the corresponding consumption quantities (from the Jiangsu Provincial Statistical Yearbook, 1991-2023), with separate urban and rural area distinctions.

Table S3 Jiangsu's virtual nitrogen factor[6]

Type/year	1998	2003	2008	2013	2018
Grain	2.48	2.50	2.37	2.39	2.31
Vegetable	2.04	2.04	2.04	1.93	2.09
Fruit	13.69	13.60	12.20	12.44	10.61
Livestock meat	6.69	5.12	5.69	5.50	5.86
Poultry	5.19	3.75	6.14	6.06	6.28
Aquatic product	2.94	3.37	2.99	2.99	3.06
Eggs	6.66	4.84	5.06	5.62	5.02
Dairy	7.52	5.53	4.83	7.34	7.59

Note: The virtual nitrogen factor (VNFs) in Table S3 (1998, 2003, 2008, 2013, 2018) is linearly interpolated for intermediate years (such as 1999-2002). As for 1990-1997 and 2018-2022, they were obtained through linear interpolation based on the overall development trend of the virtual nitrogen factor from 1998 to 2018.

Fig. S1 Changes in the ratio of food consumption nitrogen footprint and food production nitrogen footprint to total food nitrogen footprint in urban areas (a) and rural areas (b)

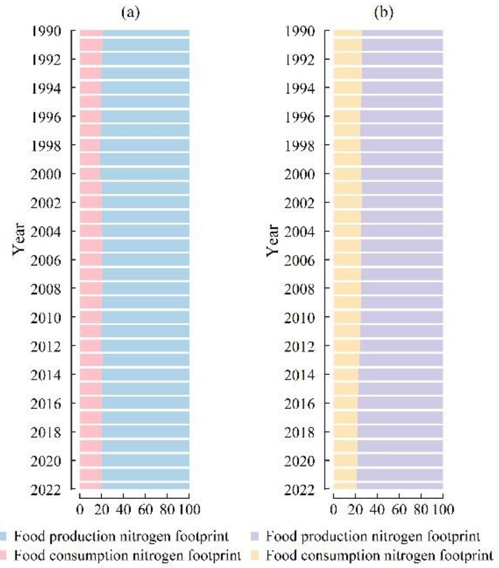

REFERENCES

1. Cui, S., Shi, Y., Malik, A., et al. 2016. A hybrid method for quantifying China's nitrogen footprint during urbanization from 1990 to 2009. Environment International, 97,137-145.

2. China Health and Nutrition Survey（1989-2015）. https://chns.cpc.unc.edu/data/datasets/.

3. Zhang, Z. 2020. Research on Chinese food waste behavior under wage growth. Nanjing Agricultural University.

4. Wang, W. 2023. Research on the Impact of Household Income on the Carbon Footprint of Food Waste in Resident Households. Shanghai University of Finance and economics.

5. Jiangsu Provincial Statistical Yearbook (1991-2023).

6. Zhai, J., Han, B., Li, H. et al., 2023. Accounting for the nitrogen footprint of food production in Chinese provinces during 1998–2018. Journal of Cleaner Production, 389, 136011.
